# The palliative effect of mulberry leaf and olive leaf ethanolic extracts on hepatic CYP2E1 and caspase-3 immunoexpression and oxidative damage induced by paracetamol in male rats

**DOI:** 10.1007/s11356-023-25152-z

**Published:** 2023-01-13

**Authors:** Hosny Abd El Fadil, Amany Behairy, Lamiaa L. M. Ebraheim, Yasmina M. Abd-Elhakim, Heba Hussein Fathy

**Affiliations:** 1 Department of Pharmacology, Faculty of Veterinary Medicine, Zagazig University, Zagazig, Egypt; 2 Department of Physiology, Faculty of Veterinary Medicine, Zagazig University, Zagazig, Egypt; 3 Department of Histology and Cytology, Faculty of Veterinary Medicine, Zagazig University, Zagazig, Egypt; 4 Department of Forensic Medicine and Toxicology, Faculty of Veterinary Medicine, Zagazig University, Zagazig, Egypt

**Keywords:** Paracetamol, Mulberry leaves, Olive leaves, Liver; Silymarin, Antioxidant enzymes, CYP2E, Caspase 3

## Abstract

This study investigated the possible protective role of mulberry leaf (MLE) and olive leaf (OLE) ethanolic extracts against paracetamol (PTL)–induced liver injury in rats compared to silymarin as a reference drug. Initially, MLE and OLE were characterized using gas chromatography–mass spectrometry (GC/MS). Then, forty male Sprague Dawley rats were divided into five groups: the negative control group orally received distilled water for 35 days, the PTL-treated group (PTG) received 500 mg PTL/kg b. wt. for 7 days, the MLE-treated group (MLTG) received 400 mg MLE/kg b. wt., the OLE-treated group (OLTG) received 400 mg OLE/kg b. wt., and the silymarin-treated group (STG) received 100 mg silymarin/kg b. wt. The last three groups received the treatment for 28 days, then PTL for 7 days. The GC–MS characterization revealed that MLE comprised 19 constituents dominated by ethyl linoleate, phytol, hexadecanoic acid, ethyl ester, and squalene. Moreover, OLE comprised 30 components, and the major components were 11-eicosenoic acid, oleic acid, phytol, and à-tetralone. MLE and OLE significantly corrected the PTL-induced normocytic normochromic anemia, leukocytosis, hypercholesterolemia, and hypoproteinemia. Moreover, the MLE and OLE pretreatment considerably suppressed the PTL-induced increment in serum levels of hepatic enzymes, including alkaline phosphatase, alanine aminotransferase, and aspartate aminotransferase. Furthermore, the PTL-induced depletion in antioxidant enzymes, including glutathione peroxidase, superoxide dismutase, and catalase, and the rise in hepatic malondialdehyde content were significantly reversed by the MLE and OLE pretreatment. Besides, MLE and OLE pretreatment significantly protected the hepatic tissue against PTL-induced DNA damage, pathological perturbations, and increased caspase 3 and CYP2E1 immunoexpression. Of note, OLTG showed better enhancement of most indices rather than MLTG. Conclusively, these findings imply that OLE, with its antioxidant and antiapoptotic capabilities, is superior to MLE in protecting against PTL-induced liver injury.

## Introduction

Paracetamol (PTL), also known as acetaminophen, is one of the most commonly prescribed analgesics and antipyretics (Britza et al. 2022). PTL is a typical remedy for headaches, mild aches, and flu. In addition, when combined with opioid analgesics, PTL can treat more severe pain, like post-operative pain and palliative care for advanced cancer patients (Popiolek and Porebski 2022). Although it is commonly considered safe and tolerable, overdose or excessive use can have serious consequences, including hematotoxicity (Yousef et al. 2010), gastric ulcers (Rainsford and Whitehouse 2006), hepatotoxicity (Cairns et al. 2019), impaired reproductive function (Pereira et al. 2020), and kidney injury (Nazir et al. 2021).

Hepatotoxicity is a common complication of PTL overdose, found in over 40% of patients with acute liver failure (Wong and Graudins 2017). PTL is easily and nearly entirely absorbed through the oral route (Bannwarth 2004). The cytochrome P450 (CYP450) enzymes, principally CYP2E1, CYP3A4, and CYP1A2, metabolize 10% of PTL to generate N-acetyl-p-benzoquinone imine (NAPQI), which is then cleared via glutathione conjugation; the lasting 90% is removed via sulfation and glucuronidation (Cheung et al. 2005). When NAPQI levels are toxic, PTL-induced hepatotoxicity occurs (Kalsi et al. 2011, McGill and Jaeschke 2013).

Numerous studies have shown that using medicinal herb products can reduce PTL-induced hepatotoxicity (Elshamy et al. 2022; Singh et al. 2022). In recent years, olive leaf extract (OLE) has played an important role in the nutraceutical and pharmaceutical industries (Berköz et al. 2021). The olive leaves (OL) have many positive health impacts, such as hypo-cholesterolemic (Jemai et al. 2009), hypoglycemic (Kontogianni et al. 2013), cardio-protective (Nekooeian et al. 2014), antihypertensive (Romero et al. 2016), immune-modulatory (Vezza et al. 2019), antioxidant, anti-inflammatory (Acar-Tek and Ağagündüz 2020), and neuroprotective (Hadrich et al. 2022). The protective effects of OLE against methotrexate-induced hepatotoxicity (Abd El-Azim 2014) and chemically induced liver cirrhosis (Al-Attar and Shawush 2015) have been demonstrated in rat models.

Mulberry leaf extract (MLE), or dried powder, has been shown to have antidiabetic, anti-atherosclerosis (Cai et al. 2016; Enkhmaa et al. 2005), anti-obesity (Ann et al. 2015), antioxidant (Lee et al. 2017), anti-inflammatory (Jeong et al. 2016), antibacterial, and antimicrobial (Chan et al. 2016) properties. Several previous studies have demonstrated that mulberry leaf (ML) powder is beneficial in lowering serum low-density lipoprotein (LDL-C) and triglyceride (TG) (Aramwit et al. 2013; Kobayashi et al. 2010). ML has been shown to lower total plasma cholesterol (TC) and raise high-density lipoprotein cholesterol (HDL-C) (Huang et al. 2018). Recently, Xv et al. (2021) reported that ML may treat hepatic damage by controlling inflammation and oxidative stress, giving a theoretical basis for mulberry leaf development as a medication against high starch–induced liver disease. Nonetheless, the potential beneficial role of ML against PTL-induced hepatic injury has not been investigated.

Silymarin, a milk thistle (*Silybum marianum*) extract, is largely considered a potent herbal medication to treat hepatotoxicity (Abenavoli et al. 2010). Silymarin’s hepatoprotective properties in PTL intoxication have previously been established (Nayak et al. 2011). Moreover, Freitag et al. (2015) verified that silymarin restored the hepatocytes’ normal function and histopathology after being aberrated by APAP. Hence, silymarin has been commonly used as a hepatoprotective reference drug to evaluate the efficacy of several plant extracts to protect against PTL-induced liver injury (Sarkar et al. 2022; Singh et al. 2022). Therefore, the present study was planned to evaluate the efficiency of OLE and/or MLE to protect against the hepatotoxicity of PTL with their biological activities compared to silymarin.

## Materials and methods

### Chemicals

Paracetamol was used in tablets containing 500 mg PTL (EL Nasr Pharmaceutical Chemicals Co. “ADWIC,” Abu-Zaabal, Egypt). Silymarin was acquired as sachets from Sedico Company (Six-October City, Egypt), each sachet containing 200 mg. All of the other compounds used in the study were of analytical grade.

### Mulberry and olive leaf extract preparations

Fresh green ML and OL of excellent quality were harvested from mulberry and olive trees cultivated in Cairo governorate farms. A botanist at the Department of Plants, Faculty of Science, Zagazig University, Zagazig, Egypt, botanically identified and authenticated the leaves. The leaves were properly washed under running tap water, then shade-dried for 5 days before being pulverized to a fine powder in an electric mixer. The powdered plant material (600 g) was extracted with 70% ethanol (100 g/L) at room temperature and then filtered with Whatman filter paper no. 1. The filtrate was evaporated to dryness using a Soxhlet evaporator producing dark green thick liquid extracts weighing 105 g for OL and 52 g for ML. The extracts were diluted in distilled water for oral administration before usage (Eidi et al. 2009; Singh et al. 2017).

### Gas chromatography/mass spectrometry (GC/MS) characterization of MLE and OLE

Gas chromatography–mass spectrometry was conducted via a Fisons GC 8000 gas chromatography attached to a Fisons MD 800 mass detector in 70 eV electron impact ionization. The range of the MS scan was 35–450 amu, and the interface temperature was 230 °C (AMU). The analysis used a fused silica OV1 capillary column as the chromatographic column (25 m × 0.25 mm i.d.). The carrier gas was helium, with a 10 mL/min flow rate. The column was kept at 60 °C for 2 min before being elevated to 170 °C with a 2 °C/min heating ramp and then remained for 3 min at 170 °C. Lastly, the temperature was raised to 250 °C using a 3 °C/min heating ramp and maintained for 120 min. At 220 °C, a split mode injection was conducted. MLE and OLE compounds were identified using mass spectra from authentic chemicals, the Wiley spectral library collection, and the National Institute of Standards and Technology library (Adams 2007).

### Animals and experimental groups

Forty Sprague Dawley rats (male, 210 ± 2.2 g) were attained from the Laboratory Animal Research Unit, Faculty of Veterinary Medicine, Zagazig University. Rats were housed in cages for 1 week before the experiment in a temperature-controlled (25.1 °C) setting with free access to food and filtered water. Experimental rats were arbitrarily distributed into five groups, each containing eight rats. The control group was given distilled water orally for 35 days. The PTL-treated group (PLTG) was orally given distilled water for 28 days, then administered PTL at 500 mg/kg b. wt. (Mowsumi et al. 2013) for an additional seven consecutive days. The MLE-treated group (MLTG) orally received 400 mg/ b. wt. (Volpato et al. 2011) for 28 days, then PLT (500 mg/kg b. wt.) for 7 days. The OLE-treated group (OLTG) orally received 400 mg/kg b. wt. (Khalil et al. 2014) for 28 days, then PLT (500 mg/kg b. wt) for 7 days. The silymarin-treated group (STG) was orally administered 100 mg/kg b. wt. (Galal et al. 2012) for 28 days, then PLT (500 mg/kg b. wt.) for 7 days. The oral medications were administered through a gastric tube. Each week, every rat was weighed, and dose quantities were calculated accordingly. The rats were closely monitored for signs of discomfort, pain, damage, distress, aberrant behavior, morbidity, and mortality during the trial.

### Blood sampling and liver tissue sampling preparations

The animals were anesthetized by intraperitoneal injection of sodium pentobarbital (100 mg/kg), 24 h after receiving their last dosage of PTL. Then, two blood samples were taken by retroorbital puncture (Parasuraman et al. 2010). For the investigation of hematological indices, one sample was put in tubes with 10% EDTA as an anticoagulant. In plain test tubes, a second sample was obtained and left to clot for 30 min at room temperature. After the sample was centrifuged at 3000 rpm for 20 min, the resultant serum was kept at 20 °C until biochemical analysis. After that, the animals were humanely euthanatized, and their livers were quickly removed and cleaned in a saline solution that was ice cold. The liver homogenate was prepared by referring to an earlier described method (Kaplan & Utiger 1978). A liver portion was homogenized in phosphate buffer saline (0.1 M PBS with pH 7.4). The homogenates were then centrifuged at 10,000 rpm for 30 min at 4 °C, and the supernatants were preserved at 70 °C until analysis of oxidative stress indices. The liver’s second half was kept in 10% neutral buffered formalin for histological and immunohistochemical analyses. The third specimen was employed for the genotoxicity comet assay.

### Hematological analysis

An automated blood cell analyzer (Hemascreen 18, Hospitex diagnostic, Italy) was used to calculate the total blood count (Erythrogam and leukogram counts) (Buttarello and Plebani 2008).

### Assessment of oxidative stress parameters

Hepatic tissue homogenates were analyzed for glutathione peroxidase (GPx) activity (Paglia and Valentine 1967). Superoxide dismutase (SOD) and catalase activities were assessed following Nishikimi et al. (1972) and Sinha (1972) protocols, respectively. Malondialdehyde (MDA) content was assessed consistent with the Ohkawa et al. (1979) method.

### Serum biochemical parameters

The serum levels of aspartate aminotransferase (AST) and alanine aminotransferase (ALT) were determined using Bio Diagnostic Company reagent kits (Giza, Egypt) following the Reitman and Frankel (1957) procedures. The alkaline phosphatase (ALP) level in serum samples was determined by the kinetic method consistent with Rosalki et al. (1993) via BioSystems kits (Barcelona, Spain).

The serum contents of total protein and albumin were measured by the reagent kits of Diamond Diagnostics, Egypt, based on the methods of Henry (1964) and Doumas et al. (1971), respectively. At the same time, the globulin level was calculated by detracting albumin from total proteins. Serum TC was estimated in line with the enzymatic technique of Allain et al. (1974). The Fossati and Prencipe (1982) method was used to determine TG colorimetrically. The HDL-C was measured by the method of Warnick et al. (1983), while LDL-C and very-low-density lipoprotein cholesterol (VLDL-C) concentrations were estimated according to the method described by Friedewald et al. (1972)’s equation as follows: VLD-C = triglycerides/5, LDL-C = TC - (TG/5 + HDL-C).

### Comet assay (single-cell gel electrophoresis)

The comet test was performed using Singh et al.ʼs (1988) protocol. In brief, 1 g of the liver sample was placed in ice-cold PBS. The earlier suspension was filtered after being agitated for 5 min. The cell suspension (100 mL) was combined with low-melting agarose (600 mL, 0.8% in PBS). On pre-coated slides, this mixture (100 mL) was dispersed. For 15 min, the covered slides were submerged in lysis buffer (0.045 M Tris–brate–EDTA (TBE), 8.4 pH, with 2.5% sodium dodecyl sulfate (SDS)) for 15 min. The slides were put into an electrophoresis apparatus (2 V/cm) with TBE buffer at 100 mA for 2 min. Ethidium bromide (EtBr, 20 mL/mL) was used to stain the slides at 4 °C. The patterns of DNA fragment migration of 100 cells per sample were analyzed with a fluorescent microscope (with 420–490 nm excitation filter) while the samples were still wet. To visualize DNA damage, the EtBr-stained DNA was viewed under a fluorescence microscope at 400 magnifications. The DNA migration length (TL), the percentage of the tailed cells (%T), and the migrated DNA percentage (%DNA) were measured using Comet 5 image analysis software of Kinetic Imaging, Ltd. (Liverpool, UK) connected to a charge-coupled device (CCD) camera to qualitatively and quantitatively evaluate the extent of DNA damage in the hepatocytes (DNA%). Finally, the tail moment (TM = TL × % DNA) was computed.

### Histopathological evaluation of the liver

Animals were euthanatized, then the caudal portion of the left lateral lobe of the liver of each animal was sampled and directly fixed in a 10% neutral buffered formalin solution. Post fixation, the tissues were processed for paraffin, sliced into 5-µm sections, stained with hematoxylin and eosin, and studied with a light microscope (Suvarna et al. 2018). A multiparametric numerical lesion scoring was performed in five non-overlapped randomly selected high power fields (HPF, 40 ×) per animal to grade the histopathological alterations. The tested histological alterations were (1) the percentages of hepatocytes manifesting vacuolar and hydropic degenerations, steatosis, pyknosis, or single-cell necrosis concerning the total numbers of hepatocytes per HPF; (2) the percentages of the area fractions of central veins, portal blood vessels, sinusoids, and necrotic areas concerning the total areas of the fields by the ImageJ software version 1.41; and (3) the frequencies of hemorrhages and leukocytic infiltrations per field. The results were demonstrated as percentages (means ± SE).

### Immunohistochemical analysis

The immunohistochemical examination included staining of the formalin-fixed paraffin-embedded 5-µm hepatic tissue sections with (1) caspase-3 (CASP3) antigen using rabbit monoclonal anti-caspase-3 (CASP3) (product code; ab184787, Abcam, Inc.) at 1/1000 dilution, and (2) cytochrome P450 2E1 (CYP2E1) using rabbit polyclonal anti-cytochrome P450 2E1 primary antibody (Cat. No. PIPA579132, Thermo Fisher Scientific, Inc.) at 500 μg/mL dilution. The staining procedures were carried out consistent with the avidin–biotin-peroxidase complex technique developed by Hsu et al. (1981). The antigen–antibody complexes were visualized by 3,3′-diaminobenzidine (DAB), and nuclei were countered and stained by Mayer’s hematoxylin. Next, the degree of immunoexpression of both biomarkers was quantified in five non-overlapped randomly chosen fixed sizes (220 × 280 µm) HPF (40 ×) per marker per animal. Snapshots of the microscopic fields were taken by an AmScope microscope digital camera at the same exposure time and magnification. The percentages of the DAB brown-stained areas’ fractions (regardless of the brown color intensity) to the images’ total areas were calculated by the ImageJ software version 1.41 via the color deconvolution plugin. The results were expressed as percentages (means ± SE).

### Statistical analysis

The Kolmogorov–Smirnov and Levene’s tests were used to assess the normality and homogeneity of variances, respectively. Data were analyzed via one-way analysis of variance (ANOVA) to identify the variation between groups where normality assumptions were met, followed by Tukey’s multiple range post hoc test for pairwise comparisons. The data are presented as the mean ± SE. There were significant differences if the *p* value was less than 0.05.

## Results

### GC–MS profile of MLE and OLE

**Fig. 1:**
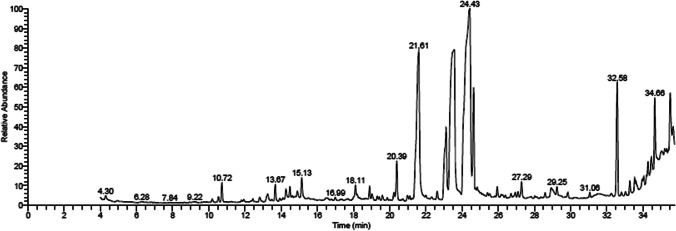
GC–MS chromatogram of mulberry leaf ethanolic extract

**Fig. 2:**
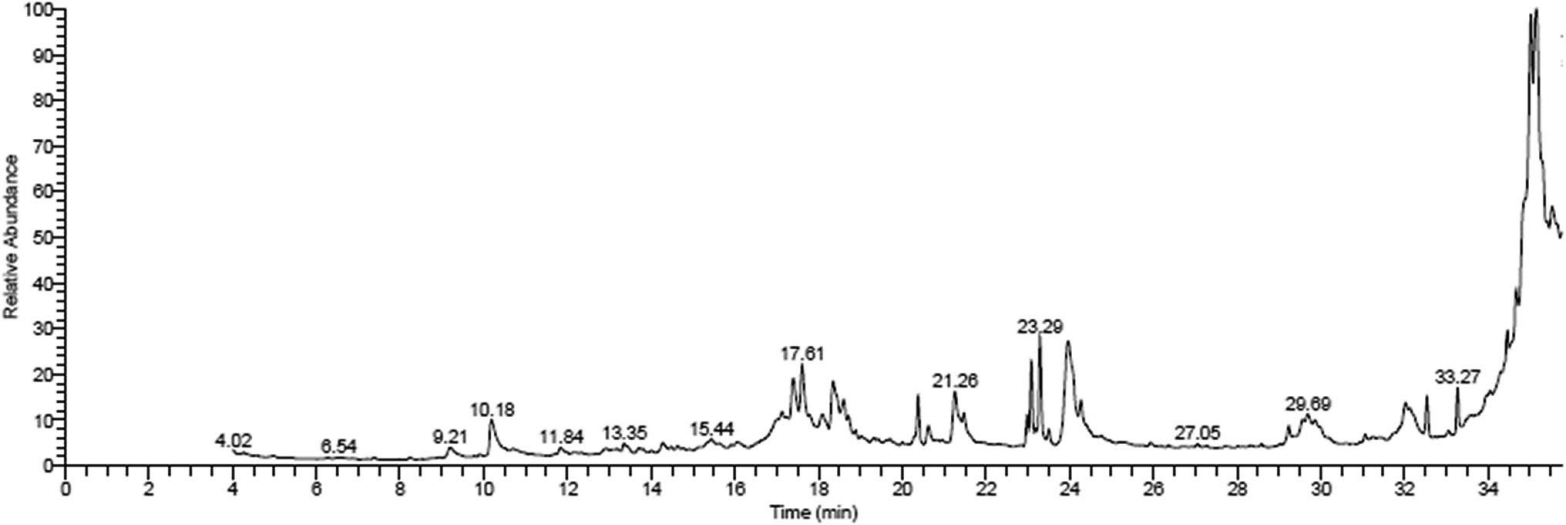
GC–MS chromatogram of olive leaf ethanolic extract

The GC–MS characterization of MLE and OLE revealed the principal constituents alongside proportional contribution to the total peak area and their retention times (Figs. 1 and 2 and Table 1). The data revealed that the MLE comprised 19 constituents, with the majority being ethyl linoleate (32.85%), phytol (20.25%), hexadecanoic acid, ethyl ester (9.81%), squalene (5.68%), linolenic acid, methyl ester (4.94%), and 9 octadecanoic acid, ethyl ester (4.44%). On the other hand, OLE comprised 30 components dominated by 11-eicosenoic acid (26.62%), oleic acid (11.54%), phytol (5.44%), cyclohepta[de]naphthalene-7,10-dione 8-hydroxy (4.55%), à-Tetralone, 8-fluoro-5,6-dimethoxy- (4.46%), and pyrrolidine-2-one-3á-(propanoic acid, methyl ester) (4.21%).

**Table 1:** Retention time (RT) and peak area (%) of the different compounds found in mulberry leaf and olive leaf ethanolic extract analyzed by GC–MS

Compound	RT (min)	Peak area %	Molecular formula	Molecular weight
a. Mulberry leaf ethanolic extract				
Ethyl linoleate	24.39	32.85	C_20_H_34_O_2_	306
Phytol	23.47	20.25	C_20_H_40_O	296
Hexadecanoic acid, ethyl ester	21.61	9.81	C_18_H_36_O_2_	284
Squalene	32.58	5.68	C_30_H_50_	410
Linolenic acid, methyl ester	23.12	4.94	C_23_H_38_O_2_	346
Octadecanoic acid, ethyl ester	24.64	4.44	C_20_H_40_O_2_	312
ç-Sitosterol	35.50	3.16	C_29_H_50_O	414
Vitamin E	34.65	2.68	C_29_H_50_O_2_	430
Hexadecanoic acid, methyl ester	20.39	1.57	C_17_H_34_O_2_	270
Isochiapin B	33.27	1.57	C_19_H_22_O_6_	346
Betulin	35.66	1.04	C_30_H_50_O_2_	442
Megastigmatrienone	15.13	0.87	C_13_H_18_O	190
ç-Tocopherol	34.27	0.72	C_28_H_48_O_2_	416
1-Eicosanol	18.11	0.68	C_20_H_42_O	298
1-Heptatriacotanol	33.96	0.64	C_37_H_76_O	536
6,8-di-c-á-Glucosyl luteolin	35.00	0.52	C_27_H_30_O_16_	610
Neophytadiene	18.89	0.48	C_20_H_38_	278
Rhodopin	35.20	0.29	C_40_H_58_O	554
Farnesyl acetone	20.23	0.23	C_18_H_30_O	262
b. Olive leaf ethanolic extract				
11-Eicosenoic acid	35.01	26.62	C_40_H_76_O_2_	588
Oleic acid	23.95	11.54	C_18_H_34_O_2_	282
Phytol	23.29	5.44	C_20_H_40_O	296
Cyclohepta[de]naphthalene-7,10-dione,8-hydroxy	17.61	4.55	C_14_H_8_O_3_	224
à-Tetralone, 8-fluoro-5,6-dimethoxy-	17.39	4.46	C_12_H_13_FO_3_	224
Pyrrolidin-2-one-3á-(propanoic acid, methyl ester)	18.34	4.21	C_16_H_25_NO_5_	311
Hexadecanoic acid	21.25	3.89	C_16_H_32_O_2_	256
Oleic acid, methyl ester	23.09	3.89	C_19_H_36_O_2_	296
(Z)-(Z)-icos-11-en-1-yl icos-11-enoate	34.82	3.37	C_40_H_76_O_2_	588
Hexadecanoic acid, methyl ester	20.38	2.58	C_17_H_34_O_2_	270
Benzoic acid, 4-formyl-, methyl ester	10.17	2.46	C_9_H_8_O_3_	164
Betulin	32.13	2.33	C_30_H_50_O_2_	442
á-Sitosterol	35.52	2.27	C_29_H_50_O	414
Lanosta-8,24-dien-3-ol, acetate, (3á)-	29.56	2.14	C_32_H_52_O_2_	468
Dotriacontane	33.27	2.12	C_27_H_56_	380
Squalene	32.54	2.07	C_30_H_50_	410
Lupeol	32.03	1.89	C_30_H_50_O	426
Vitamin E	34.66	1.83	C_29_H_50_O_2_	430
Isochiapin B	34.45	1.66	C_19_H_22_O_6_	346
à-l-Galactopyranoside, methyl 6-deoxy-2-O-(trimethylsilyl)-, cyclic butylboronate	18.10	1.47	C_14_H_29_BO_5_Si	316
6,9-Octadecadienoic acid, methyl ester	22.98	1.38	C_19_H_34_O_2_	294
Oleic acid	24.27	1.24	C_18_H_34_O_2_	282
Baimuxinal	29.23	1.09	C_15_H_24_O_2_	236
Galacto-heptulose	16.98	0.91	C_7_H_14_O_7_	210
Hexadecanoic acid, ethyl ester	21.47	0.70	C_18_H_36_O_2_	284
Heptadecanoic acid, 9-methyl-, methyl ester	23.50	0.63	C_19_H_38_O_2_	298
5,8,11,14-Eicosatetraynoic acid, TMS derivative	18.72	0.60	C_23_H_32_O_2_Si	368
6,8-di-c-á-Glucosyl luteolin	35.65	0.57	C_27_H_30_O_16_	610
Ethyl iso-allocholate	33.94	0.48	C_26_H_44_O_5_	436
1-Heptatriacotanol	13.35	0.43	C_19_H_34_O_2_	294

### Effects on body weight gain and hepatosomatic index

As displayed in Table 2, the rats orally administered PTL for 7 days exhibited a significant (*p* < 0.05) drop in final body weight and weight gain relative to the control group. On the other hand, pretreatment with MLE, OLE, and silymarin displayed a significant (*p* < 0.05) enhancement in final body weight and body gain compared to PTG. A non-significant difference was observed in final body weight and body weight gain between MLTG and OLTG. Also, a non-significant difference in final body weight and body gain was recorded between STG- and OLTG-treated groups.

**Table 2:** Effect of mulberry leaf and olive leaf ethanolic extract pre-treatments for 28 days on body weight gain and hepatosomatic index of adult male Sprague Dawley rats orally administered paracetamol for 7 days

	Control	PTG	MLTG	OLTG	STG
Initial body weight (g)	218.33 ± 0.88	220.33 ± 3.18	217.67 ± 1.45	216.67 ± 3.33	213.00 ± 4.04
Final body weight (g)	254.33^a^ ± 2.33	202.33^d^ ± 2.33	233.33^c^ ± 2.03	239.67^bc^ ± 2.60	241.67^b^ ± 1.67
Body weight gain (g)	36.00^a^ ± 3.06	− 18.00^d^ ± 1.53	15.67^c^ ± 2.85	23.00^bc^ ± 5.69	28.67^ab^ ± 3.28
Liver weight (g)	0.649^d^ ± 0.00	0.748^a^ ± 0.00	0.679^b^ ± 0.00	0.669^c^ ± 0.00	0.664^c^ ± 0.00
Hepatosomatic index	0.255^d^ ± 0.00	0.370^a^ ± 0.00	0.291^b^ ± 0.00	0.279^c^ ± 0.00	0.275^c^ ± 0.00

The data are expressed as the mean ± SE (*n* = 8). Means within the same row carrying different superscripts (a, b, and c) are significantly different (one-way ANOVA followed by Tukey’s multiple range test, *p* < 0.05)

*PTG* paracetamol-treated group, *MLTG* mulberry leaf extract–treated group, *OLTG* olive leaf extract–treated group, *STG* silymarin-treated group

Concerning the hepatosomatic index, the PTL-administered group showed a significant (*p* < 0.05) increase in hepatosomatic index relative to the control group (Table 2). On the contrary, the pre-administration of MLE, OLE, and silymarin decreased the hepatosomatic index compared to PTG. The improvement in hepatosomatic index was significantly (*p* < 0.05) higher in OTLG than in MTLG. Moreover, no significant difference was recorded in the hepatosomatic index between rats in OLTG and STG.

### Effects on erythrogram and leukogram

As shown in Table 3, rats in PTG had an obvious normocytic normochromic anemia reflected by a significant (*p* < 0.05) reduction in red blood cells (RBCs), packed cell volume percent (PCV %), and hemoglobin concentration (Hb) by 29.68%, 29.23%, and 30.15%, respectively, compared to the control group. On the contrary, oral dosing of OLE and silymarin evoked a significant (*p* < 0.05) increase in RBCs, Hb, and PCV % relative to PTG. Moreover, no significant differences were recorded between rats in OLTG and STG in RBCs and Hb %. While MLE oral dosing revealed only a significant (*p* < 0.05) increase in PCV % with non-significant improvement in RBCs and Hb % compared to PLTG. On the other hand, a non-significant change was observed in mean corpuscular volume (MCV), mean corpuscular hemoglobin (MCH), and mean corpuscular hemoglobin concentration (MCHC) among all treated groups.

**Table 3:** Effect of mulberry leaf and olive leaf ethanolic extract pre-treatments for 28 days on erythrogram and leukogram components of adult male Sprague Dawley rats orally administered paracetamol for 7 days

	Control	PTG	MLTG	OLTG	STG
Erythrogram					
RBCs (× 10^6^/μL)	7.11^a^ ± 0.15	5.00^c^** ± **0.43	5.23^c^ ± 0.32	6.13^b^ ± 0.25	6.40^ab^ ± 0.13
Hb (g/dL)	15.16^a^ ± 0.10	10.59^d^ ± 1.05	11.17^ cd^ ± 0.25	12.68^bc^ ± 0.17	13.76^ab^ ± 0.27
PCV (%)	45.37^a^ ± 0.43	32.11^e^ ± 1.06	34.77^d^ ± 0.55	38.37^c^ ± 0.63	41.43^b^ ± 0.50
MCV (fl)	63.87 ± 1.46	64.83 ± 3.90	66.87 ± 3.32	62.71 ± 2.07	64.79 ± 2.08
MCH (pg)	21.33 ± 0.31	21.15 ± 0.93	21.50 ± 1.23	20.77 ± 1.14	21.51 ± 0.66
MCHC (%)	33.41 ± 0.33	32.84 ± 2.28	32.13 ± 0.36	33.08 ± 0.93	33.21 ± 0.57
Leukogram					
WBCs (× 10^3^/μL*)*	10.99^d^ ± 0.46	15.18^a^ ± 0.25	14.52^ab^ ± 0.20	13.56^b^ ± 0.41	12.06^c^ ± 0.27
Neutrophil (× 10^3^/μL*)*	1.13^c^ ± 0.03	4.20^a^ ± 0.33	3.74^a^ ± 0.10	2.70^b^ ± 0.09	1.32^c^ ± 0.14
Lymphocytes (× 10^3^/μL*)*	9.32 ± 0.43	10.37 ± 0.65	10.39 ± 0.16	9.78 ± 0.15	10.22 ± 0.26
Monocytes (× 10^3^/μL*)*	0.51 ± 0.05	0.55 ± 0.04	0.50 ± 0.02	0.51 ± 0.06	0.47 ± 0.04
Eosinophils (× 10^3^/μL*)*	0.02 ± 0.01	0.04 ± 0.00	0.03 ± 0.00	0.04 ± 0.00	0.03 ± 0.00
Basophils (× 10^3^/μL*)*	0.02 ± 0.00	0.02 ± 0.00	0.02 ± 0.00	0.01 ± 0.01	0.02 ± 0.00

The data are expressed as the mean ± SE (*n* = 8). Means within the same row carrying different superscripts (a, b, and c) are significantly different (one-way ANOVA followed by Tukey’s multiple range test, *p* < 0.05)

*PTG* paracetamol-treated group, *MLTG* mulberry leaf extract–treated group, *OLTG* olive leaf extract–treated group, *STG* silymarin-treated group, *RBCs* red blood cells, *Hb* hemoglobin, *PCV* packed cell volume, *MCV* mean corpuscular volume, *MCH* mean corpuscular hemoglobin, *MCHC* mean corpuscular hemoglobin concentration, *WBCs* white blood cells

Regarding leukogram findings, as presented in Table 3, the total leukocyte and neutrophil counts were significantly (*p* < 0.05) increased in rats of PLTG by 38.13% and 271.68%, respectively, relative to the control group. Conversely, pretreatment with OLE and silymarin significantly (*p* < 0.05) reduced the increase in total leukocyte and neutrophil counts induced by PLTG. In comparison, the MLTG revealed non-significant changes in total leukocyte and neutrophil counts relative to PTG. On the other hand, no significant changes were recorded in lymphocyte, monocyte, eosinophil, and basophil counts among different experimental groups.

### Effect on serum levels of hepatic enzymes

As presented in Table 4, rats in PTG showed a significant (*p* < 0.05) increase in the serum levels of hepatic enzymes, including ALT, AST, and ALP, by 219.30%, 75.31%, and 113.93%, respectively, compared to the control group. Nevertheless, the PTL-induced elevation of ALT, AST, and ALP was significantly (*p* < 0.05) suppressed in MLTG (40.30%, 34.57%, and 80.88%, respectively), OLTG (19.35%, 12.35%, and 50.46%, respectively), and STG (29.03%, 9.87%, and 25.23%, respectively) compared to the control group. Of note, the reduction in ALT, AST, and ALP was significantly (*p* < 0.05) higher in OTLG than in MTLG. Moreover, no significant difference was recorded in the serum levels of ALT and AST between rats in OLTG and STG.

**Table 4:** Effect of mulberry leaf and olive leaf ethanolic extracts pre-treatment for 28 days on serum biochemical analysis and hepatic oxidative status of adult male Sprague Dawley rats orally administered paracetamol for 7 days

	Control	PTG	MLTG	OLTG	STG
Liver function					
ALT (U/mL)	20.67^d^ ± 0.67	66.00^a^ ± 1.15	29.00^b^ ± 1.73	24.67 ^c^ ± 1.45	26.67^bc^ ± 0.88
AST (U/mL)	54.00^d^ ± 2.31	94.67^a^ ± 1.45	72.67^b^ ± 1.76	60.67^c^ ± 1.33	59.33^c^ ± 0.67
ALP (u/100 mL)	38.33^e^ ± 1.20	82.00^a^ ± 1.53	69.33^b^ ± 0.67	57.67^c^ ± 1.45	48.01^d^ ± 1.15
Total protein (g/dL)	6.69^a^ ± 0.07	4.79^e^ ± 0.09	5.02^d^ ± 0.07	6.15^b^ ± 0.03	5.72^c^ ± 0.01
Albumin (g/dL)	4.23^a^ ± 0.12	2.34^e^ ± 0.07	2.56^d^ ± 0.05	3.62^b^ ± 0.03	3.29^c^ ± 0.01
Globulin (g/dL)	2.46 ± 0.05	2.45 ± 0.03	2.47 ± 0.02	2.43 ± 0.02	2.53 ± 0.03
Lipid profile					
TC (mg/ ±)	38.00^d^ ± 0.58	74.67^a^ ± 1.67	63.00^b^ ± 1.53	48.00^c^ ± 2.08	46.67^c^ ± 0.88
TG (mg/dL)	27.67^e^ ± 1.33	74.33^a^ ± 2.60	62.00^b^ ± 1.53	52.67^c^ ± 1.76	38.33^d^ ± 0.33
HDL-C (mg/dL)	27.33^a^ ± 1.20	8.33^d^ ± 0.67	14.67^c^ ± 1.86	19.67^b^ ± 1.33	26.00^a^ ± 1.53
LDL-C (mg/dL)	5.13^d^ ± 1.21	51.47^a^ ± 2.42	35.93^b^ ± 0.52	17.80^c^ ± 3.04	13.00^c^ ± 0.83
VLDL-C (mg/dL)	5.53^e^ ± 0.27	14.87^a^ ± 0.52	12.40^b^ ± 0.31	10.53^c^ ± 0.35	7.67^d^ ± 0.07
Hepatic oxidative status					
CAT (U/g)	3.99^a^ ± 0.01	1.89^e^ ± 0.07	3.35^d^ ± 0.03	3.86^b^ ± 0.03	3.75^c^ ± 0.01
SOD (U/g)	376.32^a^ ± 2.20	314.32^e^ ± 2.41	333.24^d^ ± 1.29	366.19^b^ ± 0.57	351.24^c^ ± 1.21
GPx (U/g)	3.97^a^ ± 0.00	0.77^e^ ± 0.02	1.81^d^ ± 0.00	2.91^b^ ± 0.03	2.75^c^ ± 0.02
MDA (nmol/g)	19.09^d^ ± 0.96	86.60^a^ ± 2.94	43.39^b^ ± 2.39	28.54^c^ ± 1.55	29.68^c^ ± 0.37

The data are expressed as the mean ± SE (*n* = 8). Means within the same row carrying different superscripts (a, b, and c) are significantly different (one-way ANOVA followed by Tukey’s multiple range test, *p* < 0.05)

*PTG* paracetamol-treated group, *MLTG* mulberry leaf extract–treated group, *OLTG* olive leaf extract–treated group, *STG* silymarin-treated group, *ALT* alanine aminotransferase, *AST* aspartate aminotransferase, *ALP* alkaline phosphatase, *TC* total cholesterol, *TG* triglycerides, *HDLC* high-density lipoprotein cholesterol, *LDLC* low-density lipoprotein cholesterol, *VLDLC* very-low-density lipoprotein cholesterol, *CAT* catalase, *SOD* super oxide dismutase, *GPx* glutathione peroxidase, *MDA* malondialdehyde

### Effect on lipid and protein profiles

As demonstrated in Table 4, rats in PTG showed a significant (*p* < 0.05) increase in TC, TG, LDL-C, and VLDL-C by 96.5%, 168.63%, 903.31%, and 168.90%, respectively, compared to the control group. At the same time, a significant (*p* < 0.05) reduction in the serum levels of HDL-C by 69.52% was recorded in the PTG compared to the control group. Yet, the PTL-induced rise of TC, TG, LDL-C, and VLDL-C was significantly (*p* < 0.05) repressed in MLTG (65.79%, 124.07%, 600.39%, and 124.23%, respectively), OLTG (26.32%, 90.35%, 246.98%, and 90.42%, respectively), and STG (22.82%, 38.53%, 153.41%, and 38.70%, respectively) compared to the control group. Moreover, the oral dosing of MLE, OLE, and silymarin significantly (*p* < 0.05) restored HDL-C levels by 46.32%, 28.03%, and 4.87%, respectively, compared to the control group. It is noteworthy that the improvement in lipid profile parameters, including TG, HDL-C, and VLDL-C, was significantly (*p* < 0.05) higher in OTLG than in MTLG. Moreover, no significant difference was recorded in the serum levels of TC and LDL-C between rats in OLTG and STG.

Regarding the changes in protein profile components, a significant (*p* < 0.05) reduction in the serum levels of total protein and albumin by 28.40% and 44.68%, respectively, was recorded in the PTG compared to the control group (Table 4). Nonetheless, the oral dosing of OLE, MLE, and silymarin significantly (*p* < 0.05) restored total protein and albumin levels in MLTG (24.96% and 39.48%, respectively), OLTG (8.07% and 14.42%, respectively), and STG (14.50% and 22.22%, respectively) compared to the control group. Of note, OTLG had significantly (*p* < 0.05) higher total protein and albumin than MTLG. On the other hand, no significant changes were recorded in globulin levels among different experimental groups.

### Effect on hepatic oxidative stress and lipid peroxidation indicators

As shown in Table 4, PTL oral dosing for 7 days in rats significantly (*p* < 0.05) depleted the hepatic levels of CAT, SOD, and GPx by 52.63%, 16.48%, and 80.60%, respectively, compared to the control group. On the other hand, the hepatic MDA content was significantly (*p* < 0.05) increased in PTG by 353.64% relative to the control group.

Oppositely, the oral dosing of OLE, MLE, and silymarin significantly (*p* < 0.05) restored CAT, SOD, and GPx levels in the hepatic tissues of MLTG (16.04%, 11.45%, and 54.41%), OLTG (3.26%3.26%, 2.69%, and 26.70%), and STG (6.02%, 6.66%, and 30.73%) compared to the control group. Moreover, the PTL-induced elevation of hepatic MDA content was significantly (*p* < 0.05) suppressed in MLTG, OLTG, and STG to be 127.29%, 49.50%, and 55.47% compared to the control group. Of note, OTLG had significantly (*p* < 0.05) higher CAT, SOD, and GPx but lower MDA levels than MTLG. Moreover, no significant difference was recorded in the hepatic tissue content of MDA between rats in OLTG and STG.

### Effect on DNA damage of hepatic tissue

As demonstrated in Table 5, the PTG demonstrated a significant (*p* < 0.05) increase in the comet variables, including tail DNA %, tail length, the percentage of tailed cells, and tail moment compared to the control group. On the contrary, OLE and silymarin pretreatment significantly (*p* < 0.05) reduced the PTL-induced increase in the four comet variables. While MLE pretreatment significantly (*p* < 0.05) reduced the PTL-induced increment in the percentage of tailed cells and tail moment. It is noteworthy that OTLG had significantly (*p* < 0.05) lesser tail DNA %, tail length, the percentage of tailed cells, and tail moment than MTLG. Moreover, no significant difference was recorded in the tail DNA%, the percentage of tailed cells, and tail moment between rats in OLTG and STG.

**Table 5:** Effect of mulberry leaf and olive leaf ethanolic extracts pre-treatment for 28 days on comet assay parameters of liver of adult male Sprague Dawley rats orally administered paracetamol for 7 days

	Control	PTG	MLTG	OLTG	STG
Tail length	5.56^c^** ± **0.03	9.12^a^** ± **0.45	8.33^a^ ± 0.096	6.16^c^ ± 0.24	7.03^b^ ± 0.27
%DNA in tail	6.65 ^d^ ± 0.19	21.57^a^ ± 0.69	17.46^a^** ± **0.42	8.38^bc^ ± 0.31	12.49^b^ ± 2.81
Tail moment	0.37^cd^ ± 0.017	1.61^a^ ± 0.11	0.95^b^ ± 0.14	0.48^dc^ ± 0.011	0.66^c^ ± 0.02
The percentage of tailed cells (%T)	10.53^d^ ± 0.52	18.40^a^ ± 0.38	15.17^b^ ± 0.44	11.70^ cd^ ± 0.35	12.87^bc^ ± 0.58

The data are expressed as the mean ± SE (*n* = 8). Means within the same row carrying different superscripts (a, b, and c) are significantly different (one-way ANOVA followed by Tukey’s multiple range test, *p* < 0.05)

*PTG* paracetamol-treated group, *MLTG* mulberry leaf extract–treated group, *OLTG* olive leaf extract–treated group, *STG* silymarin-treated group, *%T* the percentage of tailed cells, % DNA; percentage of migrated DNA

### Histopathological findings

**Fig. 3:**
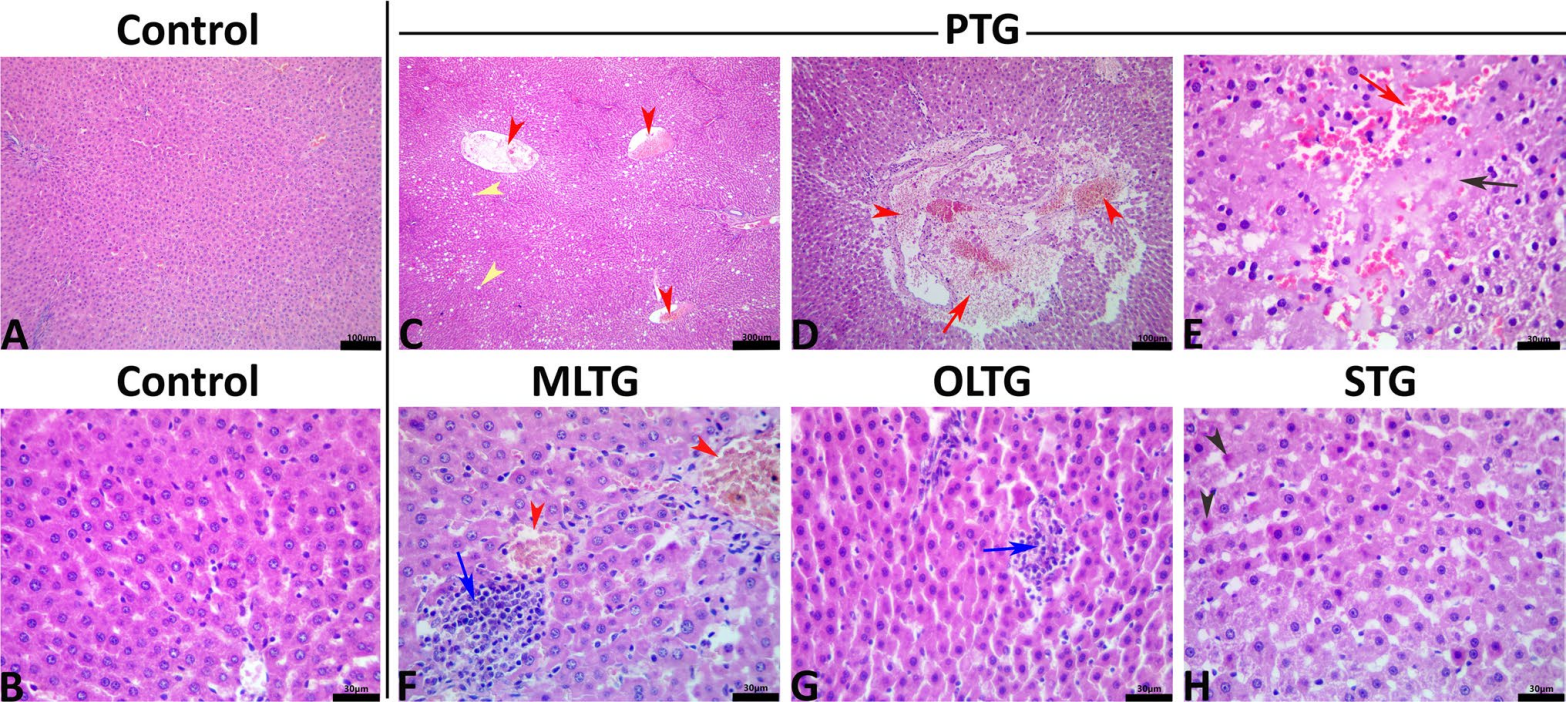
Representative photomicrograph of the H&E-stained hepatic tissue sections showing normal histological pictures in the control rats (**A** and **B**). The paracetamol-treated rat showing congestion of the central veins (red arrowheads) and steatosis (yellow arrowheads) (**C**), necrotic area replaced by blood (red arrows) and vascular congestion (red arrowhead) (**D**), coagulative necrosis of hepatocytes (black arrow) with sinusoidal dilatation (red arrow) (**E**). The MLE-treated rat showing congestion (red arrowhead) and necrotic area replaced by inflammatory cells, mostly mononuclear cells (blue arrow) (**F**). The OLE-treated rat showing a minute necrotic area replaced by mononuclear cells (blue arrow) (**G**). The silymarin-treated rat showing hepatic apoptotic cells (black arrowheads) (**H**)

The livers of the control rats showed normal histological pictures without any pathological alterations (Fig. 3(A and B)). In contrast, those of the PTG showed various degrees of hepatotoxic structural alterations manifested by congestions of the central veins and portal blood vessels, sinusoidal dilatation, vacuolar and hydropic degenerations, microvesicular and macrovesicular steatosis, portal and intralobular inflammatory cell infiltrations, and single-cell necrosis. In addition, variable degrees of centrilobular necrosis with replacement of the necrotic hepatocytes with blood and/or inflammatory cells were evident (Fig. 3(C, D, and E)). The hepatoprotective effects of MLE against the PTL-induced hepatopathy were very weak, as the hepatic tissue sections taken from the MLTG exhibited nearly the same histopathological alterations seen in the PTG but with a slight decrease in their severities (Fig. 3(F)). Interestingly, the hepatoprotective effects of OLE were notable. Although it did not fully regain the normal hepatic histology, the histological recovery was remarkable as a sharp reduction in the frequencies and severities of the PTL-induced hepatopathy alterations were seen in almost all the hepatic tissue sections of the OLTG. This group’s most frequent hepatopathy alterations were vascular congestion, sinusoidal dilatations, and intralobular small mononuclear cell foci (Fig. 3(G)). Silymarin oral dosing moderately diminished the severities and frequencies of the PTL-induced structural alterations. Yet, the hepatic parenchyma still exhibited numerous degenerative changes, particularly the cytoplasmic vacuolation, nuclear pyknosis, and single-cell necrosis (Fig. 3(H)). The multiparametric numerical lesion scoring in all groups was demonstrated in Table 6

**Table 6:** Quantitative lesion scoring and immunohistochemical expression of CASP3 and CYP2E1 in the hepatic tissue of adult male Sprague Dawley rats pretreated with mulberry leaf and olive leaf ethanolic extracts for 28 days before oral administration of paracetamol for 7 days

	Control	PTG	MLTG	OLTG	STG
Lesion and immunoexpression					
CYP2E1 (DAB area fraction)	5.78^c^ ± 0.15	14.77^a^ ± 0.55	14.31^a^ ± 0.48	6.53^c^ ± 0.33	9.36^b^ ± 0.42
CASPASE3 (DAB area fraction)	0.10^d^ ± 0.04	10.06^a^ ± 0.23	9.85^a^ ± 0.20	1.01^c^ ± 0.13	3.06^b^ ± 0.37
Central veins	1.23^e^ ± 0.041	4.51^a^ ± 0.23	3.42^b^ ± 0.18	2.06 ^d^ ± 0.18	2.65^c^ ± 0.14
Portal blood vessels	1.90^d^ ± 0.12	6.36^a^ ± 0.62	4.35^b^ ± 0.21	2.63^ cd^ ± 0.10	3.08^c^ ± 0.18
Sinusoidal spaces	4.99 ^d^ ± 0.32	10.17^a^ ± 0.30	8.55^b^ ± 0.25	5.63^ cd^ ± 0.24	6.26^c^ ± 0.31
Necrotic areas	0.00^b^ ± 0.00	7.35^a^ ± 2.73	2.42^b^ ± 0.84	0.46^b^ ± 0.25	0.72^b^ ± 0.37
Frequencies					
Vacuolar and hydropic degeneration	0.00^c^ ± 0.00	34.73^a^ ± 7.24	18.59^b^ ± 1.32	3.57^c^ ± 0.31	5.88^c^ ± 0.68
Sinusoidal spaces	4.99 ^d^ ± 0.32	10.17^a^ ± 0.30	8.55^b^ ± 0.25	5.63^ cd^ ± 0.24	6.26^c^ ± 0.31
Pyknosis	0.00^b^ ± 0.00	3.65^a^ ± 0.66	1.53^b^ ± 0.35	0.46^b^ ± 0.25	1.49 ^b^ ± 0.95
Single-cell necrosis	0.00^c^ ± 0.00	1.41^a^ ± 0.20	0.78 ^b^ ± 0.22	0.42^bc^ ± 0.22	0.79^b^ ± 0.23
Inflammatory infiltrate	0.00^d^ ± 0.00	16.00^a^ ± 5.81	8.00 ^ab^ ± 3.27	6.00 ^ab^ ± 3.06	8.00 ^ab^ ± 3.27

The data are expressed as the mean ± SE (*n* = 8). Means within the same row carrying different superscripts (a, b, and c) are significantly different (one-way ANOVA followed by Tukey’s multiple range test, *p* < 0.05)

*PTG* paracetamol-treated group, *MLTG* mulberry leaf extract–treated group, *OLTG* olive leaves extract–treated group, *STG* silymarin-treated group, *CASP3* caspase-3, *CYP2E1* cytochrome P450 2E1

### Immunohistochemical findings

**Fig. 4:**
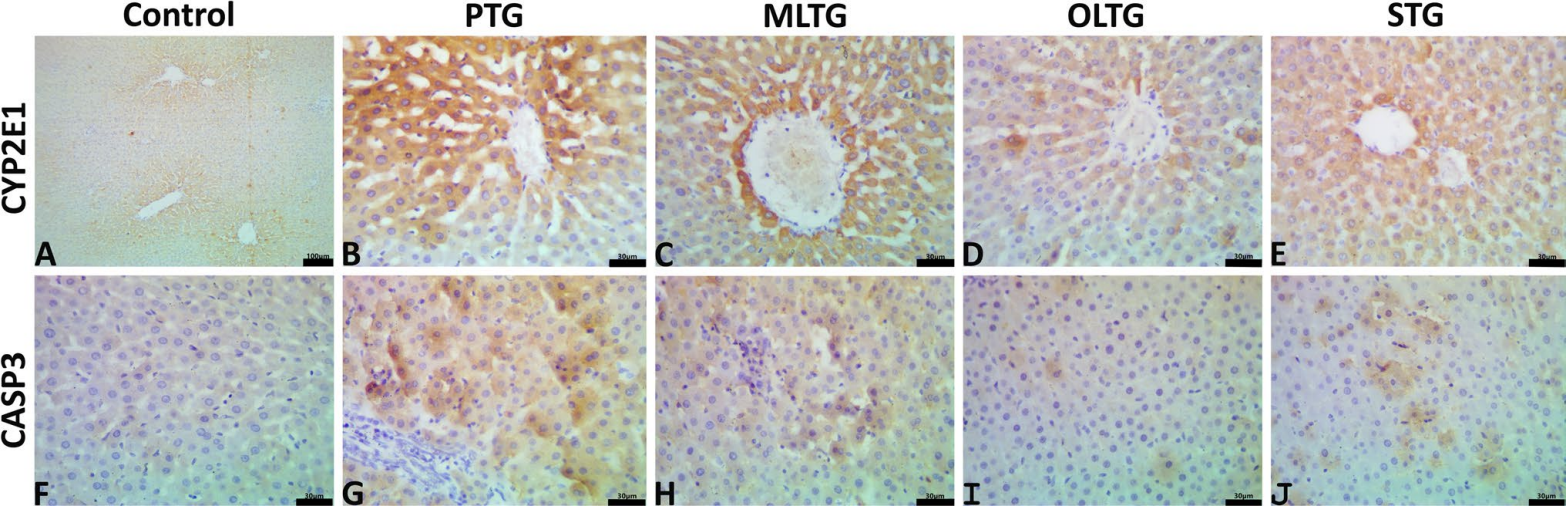
(**A**–**E**) Representative photomicrograph of the CYP2E1-stained hepatic tissue sections in control (**A**), PTG (**B**), MLT (**C**), OLT (**D**), and STG (**E**). The scale bar is 30 μm. (**F**–**J**) Representative photomicrograph of CASP3-stained hepatic tissue sections in control (**F**), PTG (**G**), MLT (**H**), OLT (**I**), and STG (**J**). The scale bar is 30 μm

The image analysis declared that PTL treatment upregulated the hepatic expression of CYP2E1 and CASP3 as the DAB brown area fractions in the livers of the PTG (14.77 ± 0.55 and 10.06 ± 0.23, respectively) (Fig. 4(B and G)), was significantly (*p* < 0.05) higher compared to the control rats (5.78 ± 0.15 and CASP3, 0.10 ± 0.04, respectively) (Table 6, Fig. 4(A and F)). On the contrary, OLE (Fig. 4(D and I)) and silymarin (Fig. 4(E and J)) pretreatment significantly (*p* < 0.05) suppressed the PTL-induced increase in the immune expression of CYP2E1 (6.53 ± 0.33 and 9.36 ± 0.42, respectively) and CASP3 (1.01 ± 0.13 and 3.06 ± 0.37, respectively). On the other hand, MLE oral pretreatment (Fig. 4(C and H)) evoked no significant alterations in the levels of expression of CYP2E1 (14.31 ± 0.48) and CASP3 (9.85 ± 0.20) compared to the PTG.

## Discussion

Currently, a global interest has been directed toward using medicinal herb products to reduce adverse effects associated with drug use (Abd-Elhakim et al. 2022, 2021a, b; Abd El-Rahman et al. 2020). The current study revealed that PTL oral dosing for 7 days resulted in a significant decrease in weight gain with a significant increase in hepatosomatic index. The weight loss with PTL medication could be linked to its metabolic effects (Alias et al. 2015). The significant increase in the hepatosomatic index is consistent with El-Gendy (2012). Pretreatment with MLE, OLE, and silymarin ameliorated the decrease in body weight and the increase in liver weight induced by PTL, possibly due to the phenolic compounds that reduce fat accumulation in the organs. For instance, 11-eicosenoic acid, a major component of OLE in the current study, has been found to have a hypolipidemic effect in rats (Yoshinaga et al. 2021). Moreover, oleic acid, the main bioactive detected by GC–MS analysis in the OLE, has been reported to play a vital role in regulating hepatic lipogenesis (Ducheix et al. 2017). On the other hand, the major components of MLE, ethyl linoleate and phytol, have been known to reduce fat accumulation (Koo et al. 2014; Peter et al. 2014). Similarly, in the Chatturong et al. (2020) study, dried mulberry fruit powder effectively reduced liver enlargement caused by a high-fat diet. Moreover, Poudyal et al. (2010) reported that OLE attenuates liver, kidney, and heart enlargement resulting from feeding high-fat diets in rats. The authors of the earlier study related the OLE effect to its efficiency in collagen and fat deposition in rat organs. Comparably, hydroxytyrosol, a polyphenol found in OL, reduced liver, kidneys, and heart enlargement in rats fed a cholesterol-rich diet (Jemai et al. 2008).

The present study revealed that PTG rats showed normocytic normochromic anemia where RBCs, Hb, and PCV % were reduced with normal MCV compared to the control group. Comparably, Dwivedi et al. (2015) verified that PTL destroys developed RBC, slows erythropoiesis, and inhibits the erythropoietin enzyme, which is released by the kidney. Due to a decrease in the oxygen-carrying capacity of blood and a reduction in the amount of oxygen given to the tissues, the Hb concentration fell. PCV value was similarly reduced in the PTG due to a reduction in blood oxygen-carrying capacity, indicating that anemia was induced. In contrast, pretreatment with OLE significantly protected RBCs against the harmful impact of PTL. In this regard, Berköz et al. (2021) confirmed the capacity of OLE to decrease lipid peroxide in the RBC membrane, which reduces hemolysis and prolongs their life span. Moreover, Banerjee et al. (2020) verified the high efficiency of oleic acid, a major component of OLE based on GC–MS analysis, in safeguarding the morphology, intracellular antioxidant status, and the metabolic enzyme activities of erythrocytes via its antioxidant mechanisms. Furthermore, the higher amount of bioactive compounds like phytol in both OLE and MLE, with their high antioxidant capacity, could be responsible for counteracting PTL-induced normocytic normochromic anemia (P Costa et al. 2016; Santos et al. 2013; Usman et al. 2021).

There was a significant increase in total leukocyte counts and neutrophils in the PTG compared to the control group, with no significant difference in other leukocyte cells. The PTL-induced leukocytosis and neutrophilia could be related to stress, combined with inflammatory changes in tissue responsible for the phagocytosis of toxic substances. These findings agree with the previous findings of Matić et al. (2016). Acute inflammation and leukocytosis, primarily increased numbers of neutrophils, have occurred due to PTL toxic effects, which increase endothelial adhesion and promote oxidative burst (Cover et al. 2006). On the other hand, the OLTG exhibited a significant improvement in leukocyte count compared to PTG. OLE’s earlier reported anti-inflammatory role could be responsible for suppressing leukocyte production (Al-Quraishy et al. 2017). In this regard, oleic acid, the main bioactive detected by GC–MS analysis in the OLE, has been reported to have anti-inflammatory effects that were directed to attenuate inflammation in several physiological and pathological conditions by controlling the production of inflammatory mediators (Chen et al. 2019; Pegoraro et al. 2021). Moreover, oleic acid has been reported to reduce neutrophil influx into inflammatory sites (Cardoso et al. 2011).

The PTL-induced hepatic damage was evident in the current study, with a significant increase in AST, ALT, and ALP in PTG compared to the control group and histopathological results. The PTL-induced hepatic damage is mainly related to the depletion of the antioxidants, as evident here, following the NAPQI accumulation, which covalently binds to cellular proteins and consecutively increases the formation of reactive oxygen species promoting cell death (Hinson et al. 2010). Hepatocyte damage probably has led to the leak of these enzymes into the blood, which signifies hepatotoxicity (Ikponmwosa-Eweka and Eromosele 2019, Yousef et al. 2010). On the contrary, MLE, OLE, and silymarin pretreatment significantly limit hepatic enzyme leakage following PTL administration. Similarly, MLE and OLE combated the enzyme leakage induced by other hepatotoxicants (Baradaran et al. 2019; Elgebaly et al. 2018; Majid et al. 2020; Teksoy et al. 2020). The MLE and OLE could protect the hepatocytes against PTL-induced injury via the free radical scavenger and antioxidant activities of MLE and OLE constituents, especially oleic acid (Guo et al. 2019), ethyl linoleate (Ghanem et al. 2015), squalene, hexadecanoic acid (Sudha et al. 2013), phytol (Santos et al. 2013), vitamin E (Kumar et al. 2010), and hexadecanoic acid (Uma Maheswari and Reena 2017). Moreover, some OLE’s bioactive has been reported to have potent hepatoprotective activity. For instance, oleic acids ameliorated hepatocellular lipotoxicity both in vitro and in vivo by inhibiting endoplasmic reticulum stress and pyroptosis (Zeng et al. 2020). Also, phytol showed significant hepatoprotective activity in the ethanol-induced pharmacological animal model (Gupta et al. 2019).

Compared to the control group, oral dosing of PTL significantly lowered serum total protein and albumin levels, but non-significant changes in globulin were seen in PTG. Comparable findings were previously reported by Datta et al. (2013) and Mowsumi et al. (2013). On the contrary, OLE and silymarin reduced total protein and albumin alterations, but rats given MLE had only a minor protective effect. These protective properties are probably linked to the antioxidative action of the phenolic and flavonoid molecules. Similarly, Al-Janabi et al. (2013) showed that the OLE administration enhanced the albumin and total protein levels of streptozotocin-induced diabetic rats. Moreover, in irradiated rats, silymarin increases total protein (Mahmoud et al. 2020).

In the current study, PTG had significantly higher TG, TC, LDL-C, and VLDL-C levels but a significantly lower HDL-C level than the control group. The PTG-induced hyperlipidemic condition has been earlier reported by Madi Almajwal and Farouk Elsadek (2015). In this regard, PTL has been reported to reduce lipase activity, leading to a decrease in TG hydrolysis and injury to hepatic parenchymal cells, resulting in lipid metabolic disturbances in the liver (Dwivedi et al. 2015). On the contrary, MLE, OLE, and silymarin pretreatment considerably reduced TG, TC, LDL-C, and VLDL-C levels while dramatically increasing HDL-C. Similarly, anti-hyperlipidemic activity has been linked with MLE, OLE, and silymarin administration (Afify et al. 2018, Huang et al. 2018, Mahmoud et al. 2020). The earlier effect could be ascribed to their active ingredients. For instance, according to the GC–MS analysis here, 11-eicosenoic acid, the predominant component of OLE, has been known for its TG- and TC-lowering effects (Mori et al. 2000; Yoshinaga et al. 2021). Moreover, oleic acid, a chief bioactive identified by GC–MS analysis in OLE, has been reported to inhibit cholesterol and fatty acid synthesis by controlling the activity of key enzymes of fatty acid biosynthesis and cholesterologenesis (Priore et al. 2017). Also, MLE’s major constituents, like ethyl linoleate and phytol, have been reported to reduce fat accumulation (Koo et al. 2014; Peter et al. 2014). Moreover, several reports confirmed that squalene, one of the MLE bioactives as revealed by GC–MS analysis, could be effective in lowering TC and LDL-C via inhibiting hepatic hydroxymethylglutaryl-CoA reductase in the liver and downregulating the conversion from acetyl CoA to cholesterol (Ibrahim et al. 2020; Strandberg et al. 1989). Other minor components in OLE and MLE have been reported to have anti-atherosclerosis, anti-oxidation, and blood lipid control properties, especially betulin (Abdelhamid et al. 2015), hexadecanoic acid (Sudha et al. 2013), and vitamin E (Bordoloi et al. 2021).

In this investigation, PTL administration resulted in increased hepatic lipid peroxidation and a substantial decrease in hepatic SOD, CAT, and GPx activities, indicating a reduction in antioxidant capacity. PTL promoted oxidative stress and changes in endogenous antioxidant enzyme activity in an earlier study in rats (Madkour and Abdel-Daim 2013). In contrast, pretreatment with MLE, OLE, and silymarin significantly reduced PTL-induced oxidative stress in rats. In OLTG, silymarin, and MLTG, there was a significant decrease in lipid peroxidation but an increase in SOD, CAT, and GPx. The antioxidant properties of OL or ML may be due to their active components, including oleic acid (Elaiyaraja and Chandramohan 2018), phytol (Usman et al. 2021), vitamin E (Kumar et al. 2010), squalene, hexadecanoic acid (Sudha et al. 2013), and n-hexadecanoic acid (Uma Maheswari and Reena 2017). Moreover, phytol showed a strong antioxidant effect in vitro by removing hydroxyl radicals and preventing the formation of thiobarbituric acid reactive substances (Santos et al. 2013).

OLE’s capacity to chelate metal ions, including Fe and Cu, which stimulate free radical production reactions, may help to prevent the development of free radicals (Andrikopoulos et al. 2002) and by the inhibition of many inflammatory enzymes, such as lipoxygenases (de la Puerta et al. 1999). Silymarin may have antioxidant capabilities in various ways, including directly scavenging free radicals, blocking free radical production, and activating multiple antioxidant enzymes through transcription factor activation (Antika and Dewi 2021).

The frequency of tail length, tailed nuclei, percentage of DNA in the tail, and tail moment in the liver significantly increased after PTL administration, demonstrating DNA damage and apoptosis. This is consistent with prior research that found PTL genotoxic (El Morsy and Kamel 2015, Oshida et al. 2008). The covalent binding of NAPQI to mitochondrial protein could be responsible for the observed rise in tail % DNA in mouse liver and kidney (Diab and Fahmy 2020). Such interaction causes the mitochondrial cell membrane to permeabilize and lyse, culminating in the release of endonuclease G from mitochondria which then translocate to nuclei and fragment nuclear DNA (McGill et al. 2012). On the contrary, MLE and OLE pretreatment markedly reduced DNA damage, possibly due to active components that scavenged NAPQI before damaging macromolecules and generating oxidative stress in liver tissues. The histopathological findings are consistent with our biochemical and oxidative stress findings. Congestions of the central veins and portal blood arteries, sinusoidal dilatation, vacuolar and hydropic degenerations, microvesicular and macrovesicular steatosis, portal and intralobular inflammatory cell infiltrations, and single-cell death signify the harmful impact of PTL on the liver. These alterations in the cellular structure were caused by oxidative activity, which resulted in lipid peroxidation, which caused cell membrane injury. The antioxidant and hepatoprotective actions of OLE and silymarin improved the histological architecture of tissue, with very mild hepatoprotective benefits for MLE.

PTL enhanced the expression of activated CASP3 in liver cells in the current investigation, implying that activated CASP3 is involved in the intrinsic mechanism of apoptosis generated by PTL. These findings are consistent with those of other researchers who found high levels of active CASP3 in hepatocytes following PTL administration (Choi et al. 2013; Kon et al. 2007). Compared to the PTL-administered group, pretreatment with OLE and silymarin reduced apoptotic events in the liver, as seen by lower hepatic CASP3 expression. Similar antiapoptotic effects of OLE and silymarin have been documented earlier (Osman and Tantawy 2017, Teksoy et al. 2020). Several bioactives in OLE and MLE have been known by their antiapoptotic activity. For instance, oleic acid prevented apoptotic cell death in several cell lines (Ahn et al. 2013; Yamasaki et al. 2008). Also, Sakthivel et al. (2019) reported that phytol mitigated benzo(a)pyrene-induced lung carcinogenesis in Swiss albino mice through its antioxidant and antiapoptotic activity.

The most active CYP450 in catalyzing the conversion of PTL to hepatotoxic NAPQI is thought to be CYP2E1 (Bessems and Vermeulen 2001). According to the current data, the OLE efficiently reduced NAPQI generation, as evidenced by the decrease of CYP2E1 immunoexpression, and protects the liver against PTL hepatotoxicity. The potential of silymarin to alter the activity of CYP2E1 could explain its protective action against PTL-induced hepatotoxicity (Papackova et al. 2018).

## Conclusion

Finally, the findings of this study showed that OLE is a more efficient hepatoprotective agent than MLE against PTL-induced liver cell damage. The antioxidant and antiapoptotic properties of the two extracts may explain their protective impact. The current findings could lead to the developing a therapeutic cure to prevent or alleviate some of the PTL-induced side effects using natural products.

## Data Availability

All data generated or analyzed during this study are included in this published article.

## Abbreviations

ALP: Alkaline phosphatase
ALT: Alanine aminotransferase
AST: Aspartate aminotransferase
CYP2E1: Cytochrome P450 2E1
CYP450: Cytochrome P450
GC/MS: Chromatography–mass spectrometry
GPx: Glutathione peroxidase
Hb: Hemoglobin concentration
HDL-C: High-density lipoprotein cholesterol
LDL-C: Low-density lipoprotein
MCV: Mean corpuscular volume
MCH: Mean corpuscular hemoglobin
MCHC: Mean corpuscular hemoglobin concentration
MDA: Malondialdehyde
MLE: Mulberry leaf ethanolic extract
NAPQI: N-Acetyl-p-benzoquinone imine
OLE: Olive leaf ethanolic extract
PCV: Packed cell volume percent
PTL: Paracetamol
RBCs: Red blood cells
SOD: Superoxide dismutase
TC: Total cholesterol
TG: Triglyceride
